# Learning Is Better with the Hands Free: The Role of Posture in the Memory of Manipulable Objects

**DOI:** 10.1371/journal.pone.0159108

**Published:** 2016-07-14

**Authors:** Léo Dutriaux, Valérie Gyselinck

**Affiliations:** 1 Laboratoire Mémoire et Cognition, Université Paris Descartes, Sorbonne Paris Cité, Paris, France; 2 Institut de Psychologie, Centre Henri Piéron, Boulogne Billancourt, France; 3 Centre de Psychiatrie et Neurosciences, INSERM U894, Paris, France; 4 IFSTTAR—LPC, Versailles, France; UCLA, UNITED STATES

## Abstract

Grounded cognition proposes that memory shares processing resources with sensorimotor systems. The aim of the present study was to show that motor simulation participates in the conceptual representation of manipulable objects in long-term memory. In two experiments, lists of manipulable and nonmanipulable objects were presented. Participants were instructed to memorize the items while adopting different postures. In the control condition, they had to keep their hands at rest in front of them. In the interference condition, participants had to keep their hands crossed behind their back to make their hands less free for action. After each list, participants had to perform first a distractive task, and then an oral free recall. The results showed that the interfering posture produced a specific decrease in the recall of manipulable objects, but not of nonmanipulable ones. This decrease was similar when the items were presented as pictures (Experiment 1) or as words (Experiment 2), thus excluding a purely visual effect. These results provide strong evidence that the motor simulation plays a role in the memory trace of the object.

## Introduction

According to grounded-cognition theories, semantic memory shares processing resources with perception, emotion and action [1–3]. Glenberg [4] underlined the importance of the motor system, claiming that the main function of conceptual knowledge is to support action. He proposed that memory encodes the environment in terms of affordances. The affordance of an object is defined here as the potential actions that an organism can perform on it, given the actual state of the object, the prior history of the organism’s interactions with this object, and the organism’s ability to act.

Consistently, numerous studies have shown that action-related concepts involve an automatic activation of the motor cortex, analogous to when the action is actually performed (for a review, see [5]). This phenomenon, often referred to as motor simulation, has been reported during the observation of actions (e.g., [6,7]), or while processing action sentences (e.g., [8–11]), but also while processing manipulable objects. Evidence for motor simulation of affordance comes from neurophysiological studies on monkeys. The so-called “canonical” neurons, found in the F5 area of the macaque premotor cortex, fire both when the monkey simply views an object, and when the monkey actually grasps that object [12]. It has also been consistently shown in humans that both viewing manipulable objects (e.g., [13–16]) and reading the names of manipulable objects (e.g., [17–19]) activate motor regions more than nonmanipulable objects or animals. In the same vein, using Transcranial Magnetic Stimulation (TMS), several studies have shown that Motor Evoked Potentials are modulated by pictures of manipulable objects [20–22].

On the behavioral side, it has been demonstrated via the stimulus-response compatibility paradigm that the viewing of a manipulable object potentiates motor actions associated with the use of that object (e.g., [18,23–28]). Tucker and Ellis [27] asked their participants to indicate whether an object was a man-made or a natural object. The response could be either a power or a precision grip, and was performed while simultaneously viewing a real object which would normally be grasped using either a power or precision grip. Faster reaction times were found when the response and the object grip were congruent (i.e., faster for power response/power grip and precision/precision trials than power/precision and precision/power trials). In the same vein, viewing tools facilitates the gesture compatible with their grasping or their use [23]. Importantly, both studies found the same kind of results with words denoting the same manipulable objects. This suggests that this effect of object affordance results, at least in part, from semantic information in memory, rather than only from perceptual information elicited by pictures.

However, while the above-mentioned studies suggest that the processing of action-related concepts involves a motor activation, such activations may be incidental to the activation of their representations from long-term memory, rather than part of it. Therefore, these results are not only consistent with the view that conceptual and motor systems share processing resources, but also with a modular view of these two systems, as has been largely discussed by Mahon and Caramazza [29]. Consistent with the former view, there is some evidence suggesting that manipulability is a semantic dimension [30], and that conceptualization relies on action relevant properties [31,32]. As suggested by Mahon and Caramazza [29], another convincing way to show that memory and action share processing resources would be to see whether the suppression or the impairment of the motor system involves a deficit in memory.

Several studies have addressed the effect of motor interference on the processing of manipulable objects. First, it seems that using TMS on the right inferior parietal lobule, which is known for its role in praxis, slows down the naming of manipulable objects, but not the naming of nonmanipulable objects [33]. Importantly, a few neuropsychological studies confirm these results, demonstrating that brain injured patients suffering from apraxia show a decrease in manipulation knowledge ([34,35], but see [36,37]). Similarly, Witt, Kemmerer, Linkenauger and Culham [38] found that squeezing a ball in one hand made it more difficult for healthy participants to name tools whose handles faced the squeezing hand (see also [39], but see [40]). Yee, Chrysikou, Hoffman, and Thomson-Schill [41] found that a task involving hand motions interferes with the categorization of words denoting frequently manipulated objects, but not with rarely manipulated objects. An interpretation of these results is that that the retrieval of knowledge about manipulable objects from long-term memory involves the retrieval of motor information, which is impaired by a motor interference and leads to a decrease in observed performances.

An increasing amount of research has recently explored more directly the effect of motor affordance on memory (for a review, see [42]). Most of the studies focused on short-term memory with a motor interference paradigm and did not show clear-cut results [43–47]. Few experiments have tried to assess the effect of motor affordance with a long-term memory paradigm. Apel, Cangelosi, Ellis, Goslin and Fischer [48] presented auditory instructions asking participants to move the picture of objects with handles oriented to the left or the right side. When the presentation of the instructions ended, participants had to actually perform the actions described by the instructions. Results showed that right-handers remembered more instructions when the picture presented an object’s handle oriented to the right, and when the actions had to be performed with the right hand, denoting again a kind of compatibility effect. In the same vein, Pezzulo, Barca, Bocconi et Borghi [49] found that expert rock climbers had a better memory of a difficult path on a climbing wall than beginners, but that an easy path was equally well remembered in both groups. This is presumably because the greater motor repertoire of the expert climbers allowed better motor simulations. However, rather than being exclusively linked to an automatic motor simulation of affordance, these results can also be explained by a motor or a visual imagery strategy. For instance, expert climbers may be better able to image the movements needed to climb the wall. As outlined before, a more convincing way to show that long-term memory and action share processing resources would be to see whether the suppression or the impairment of the motor system involves a deficit in memory [29].

Therefore, the aim of the present work was to provide strong evidence that long-term memory and the motor system do share resources, by interfering with the motor simulation of affordance during the learning of manipulable objects. For this purpose, in two experiments, participants had to memorize manipulable or nonmanipulable objects while adopting different postures. In the control condition, they had to keep their hands at rest in front of them. In the interference condition, participants had to keep both hands behind their back (see [50,51]). After each list, participants had to perform a letter matching task, and then an oral free recall. The idea was that the interfering posture would make participants’ body less available for manual action. If, as Glenberg [4] proposed, the world is encoded in terms of affordances which are dependent on the organism’s ability to act, constraining postures should interfere with the affordance. Therefore, this should decrease the subsequent recall of manipulable objects, but not the recall of nonmanipulable objects. The effect of the constraining posture on picture memory was first examined (Exp 1). Then, to confirm that the observed effect was not solely perceptual but at least partly semantic, the same experiment was conducted with words (Exp 2).

## Experiment 1

### Method

#### Ethical statement

Ethical approval for the study was granted by the Paris Descartes University committee. All procedures performed in this study were in accordance with 1964 Helsinki declaration and its later amendments as well as comparable ethical standards.

Participants

Thirty-five right-handed undergraduates studying psychology at Paris Descartes University took part in the experiment for course credits (26 women, 9 men, mean age = 20.5 years, SD = 2.62). All participants gave their written informed consent to the experimental procedure.

#### Material

**Fig 1:**
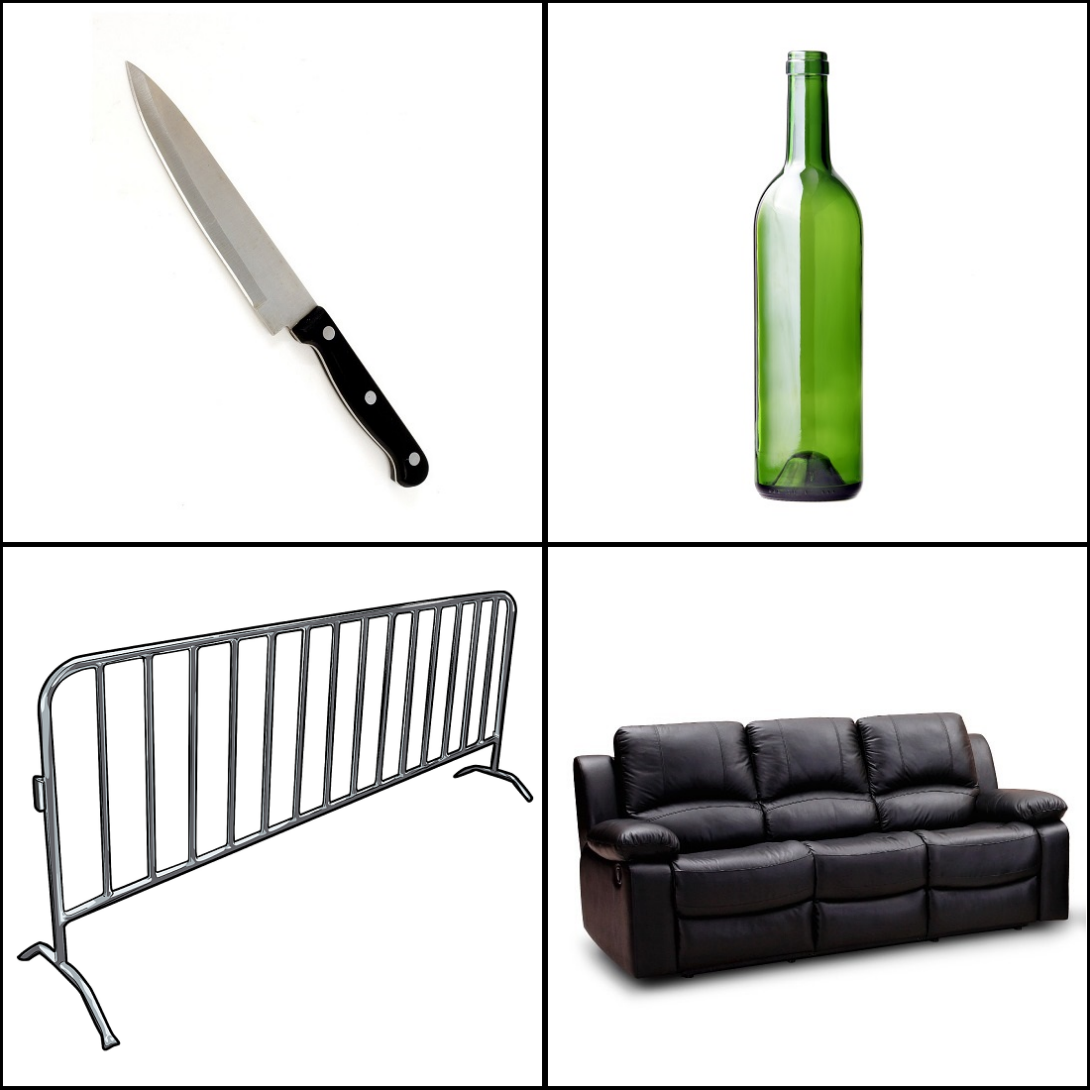
Examples of the kind of pictures used in Experiment 1. knife, bottle, fence and couch.

The pictures of 36 manipulable objects (i.e., tools, e.g., pen, hammer), and 36 nonmanipulable objects (e.g., carpet, antenna) were distributed in six lists of 12 pictures (see Fig 1 for examples). The names referring to the manipulable and nonmanipulable objects did not differ in word length (*t* < 1), objective (*t*(70) = 1.68, p = .10), or subjective frequencies (*t*(70) = 1.56, p = .12). Objective frequency means were computed with the LEXIQUE database [52], and the subjective frequency means were computed with a combination of three norms [53–55]. Objective frequencies are computed based on the frequency of each word in a representative corpus, while subjective frequencies are computed based on the results of experiments during which participants have to evaluate the frequency of each word on a Likert scale. Even though these measures are strongly correlated, we wished to ensure that frequency, which is a pervasive factor in memory and psycholinguistic studies, was controlled between lists. Each list contained six manipulable objects and six nonmanipulable ones. The stimuli were colored photographs of objects taken from internet, presented on a white background (10 cm x 10 cm, 9.5° of visual angle from a distance of around 60 cm). Pictures of the manipulable objects selected present the graspable part of the object oriented toward the dominant hand of the participants.

#### Procedure and design

Participants were tested individually in a quiet room with the experimenter present. For each list, participants were exposed to a learning phase, then to a distractive task, and finally to an oral free recall. During the learning phase, each picture was presented on a computer screen (the viewing distance was about 60cm) during 1 s, with an inter-stimulus interval of 500 ms. Participants had to memorize the items while adopting different postures. In the control condition, they were instructed to put their hands on the desk in front of them, at rest. They were told to feel free to move their hands if necessary (e.g., to scratch their head), but not to cross their arms. In the interfering condition, they had to put their hands behind their back, while holding one of their wrists with the other hand. Lists were presented in a new random order for each participant, and, within each list, pictures were presented randomly. Each participant was presented with a block of three lists in the control condition, and a block of three lists in the interfering condition. Given the randomization, the lists assigned to a block were never the same depending on the subjects. The order of the two posture blocks was counterbalanced across participants. At the beginning of the 1 minute distractive task introduced to test long-term memory effects, participants in the interfering condition had to put their hands back on the desk. During this task, pairs of letters were presented, one letter in upper case, the other in lower case, and participants had to say orally whether the letters were the same (e.g., “A”, “a”) or different (e.g., “A”, “b”). A perceptual task was preferred to a more classical counting or verbal task to avoid any semantic interference. Finally, still with their hands free at rest, they had to recall orally as many pictures as possible, without any time limit. The recall ended when the participants indicated that they had nothing left to recall.

### Results and discussion

**Fig 2:**
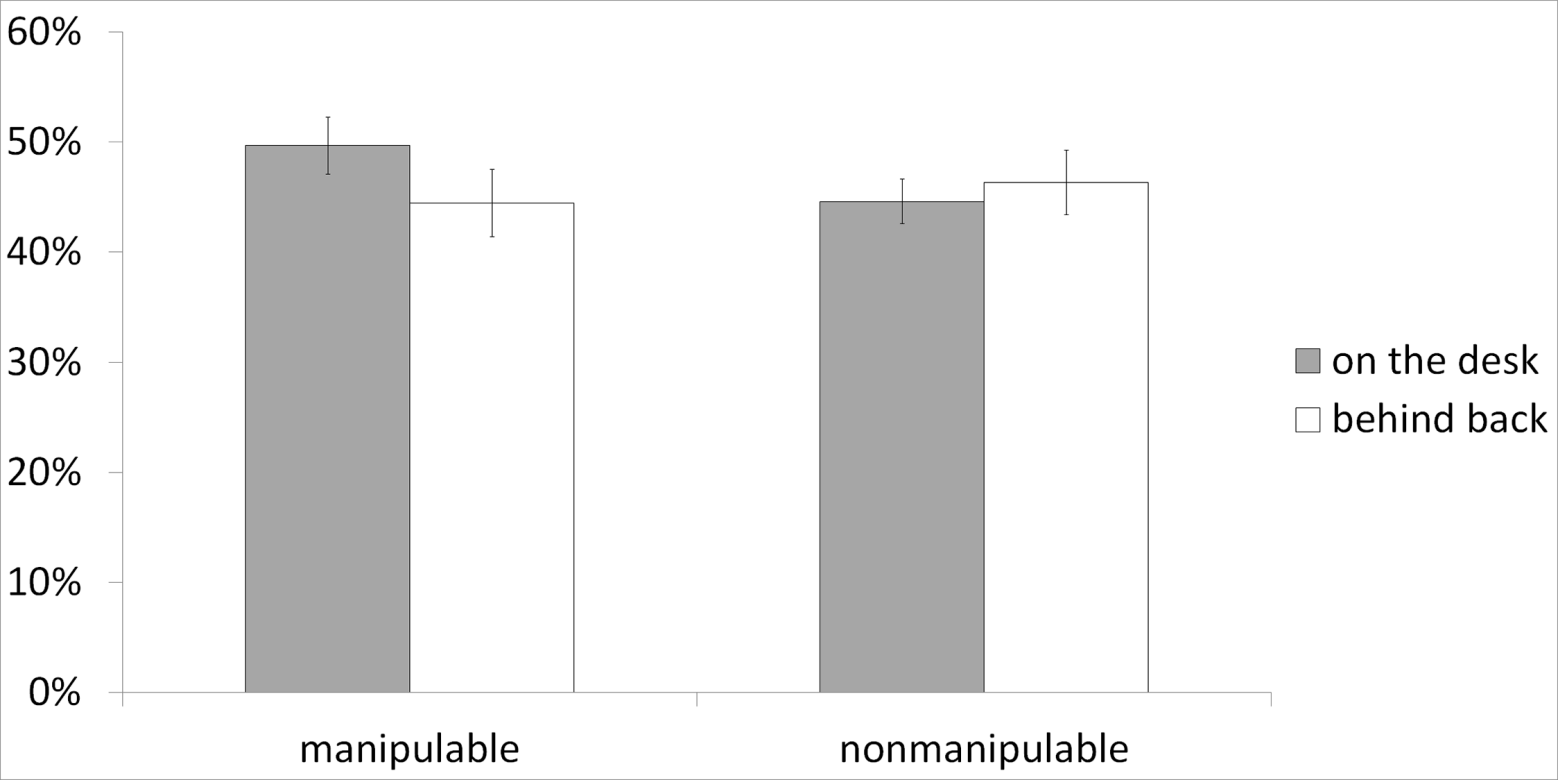
Means for the percentage of items recalled as a function of Manipulability and Posture in Experiment 1. Error bars represent standard errors of the mean.

Fig 2 shows the mean percentages of words recalled. The data were analyzed using a 2 x 2 repeated measure ANOVA, with manipulability and posture as within-subject variables. The results showed no main effect of manipulability or posture (*F*s < 1). Crucially, the expected interaction between posture and manipulability was found (*F*(1,34) = 5.76, p = .02, η_p_² = .14). As predicted, the planned comparison showed that the interfering posture decreased the recall of manipulable objects (*F*(1,34) = 5.40, p = .03) but not of nonmanipulable objects (*F* < 1).

These results clearly show that when the body is not available to act during encoding, subsequent recall of manipulable objects decreases, suggesting that motor information is part of the manipulable object memory trace. Hence, this experiment shows, as expected, that a posture constraining the hands provokes a specific decrease in recall of manipulable objects. It suggests that memory of manipulable objects and the motor system share processing resources. However, two types of affordances, perceptual and conceptual, should be considered. Perceptual affordances are the affordances conveyed by the perceptual properties of the object (e.g., shape, size, orientation), and can be used to grasp the object. Conceptual affordances are affordances conveyed by the conceptual knowledge about the object accumulated over time. Conceptual affordances include mainly motor information about the function of the object [56]. The question then arises whether putting one's hands behind one's back interferes with the motor affordance linked to the visual structural properties of the object, or with the motor affordance linked to the conceptual knowledge about the object. In other words, is the effect driven by conceptual knowledge, or solely by the visual properties of the objects? To investigate this question, a second experiment was run, similar to the first one except that object names were used instead of pictures. If the previous results are explained by conceptual knowledge, the same pattern of results should be obtained with words, whereas if it is a perceptual effect, then no interference should be observed.

## Experiment 2

### Method

#### Ethical statement

Ethical approval for the study was granted by the Paris Descartes University committee. All procedures performed in this study were in accordance with 1964 Helsinki declaration and its later amendments as well as comparable ethical standards.

#### Participants

Thirty-four right-handed undergraduates studying psychology at the Paris Descartes University took part in the experiment for course credits (26 women, 8 men, mean age = 22.26 years, SD = 6.63). All participants gave their written informed consent to the experimental procedure. None had participated in Experiment 1.

#### Material, procedure and design

The lists of objects were the same as in Experiment 1, except that words were used instead of pictures (for the list of the words, see Appendix A and B in S1 File). Material was further controlled for imageability, which did not differ between manipulable and nonmanipulable objects (*t*(70) = 1.30, p = .20) [53–55]. Words were presented on a grey background (arial 100) in the center of the screen. Apart from that, the procedure was identical to that of the Experiment 1.

### Results and discussion

**Fig 3:**
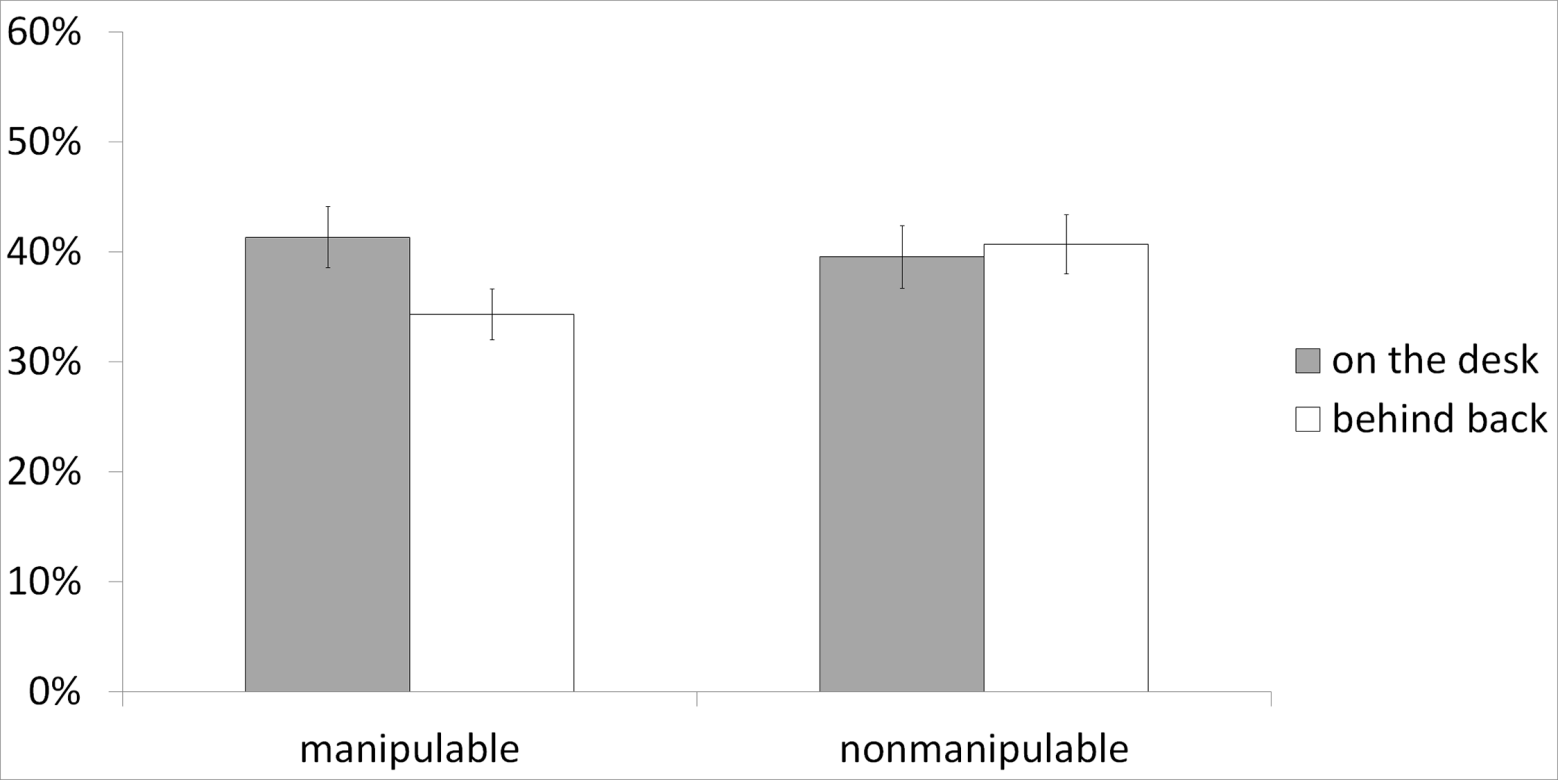
Means for the percentage of items recalled as a function of Manipulability and Posture in Experiment 2. Error bars represent standard errors of the mean.

Fig 3 shows the mean percentages of words correctly recalled. The data were analyzed using a 2 x 2 repeated measure ANOVA, with manipulability and posture as within-subject variables. The results showed no main effect of manipulability (*F*(1,33) = 1.43, p = .24) or posture (*F*(1,33) = 1.88, p = .18). Importantly, an interaction between posture and manipulability was found (*F*(1,33) = 4.30, p = .046, η_p_² = .12). The planned comparison showed that the interfering posture decreased the recall of manipulable (*F*(1,33) = 7.85, p = .008) objects but not of nonmanipulable objects (*F* < 1).

In order to compare the effects obtained on words and pictures, the data of Experiments 1 and 2 were analyzed together with a 2 x 2 x 2 mixed ANOVA, with manipulability and posture as within-subject variables, and format of presentation as a between-subject variable. Here again, neither a main effect of manipulability (*F* < 1) nor a main effect of posture (*F*(1,67) = 2.48, p = .12) were found. The main effect of format of presentation was significant (*F*(1,67) = 6.83, p = .01, η_p_² = .09), that is, recall for pictures was better than the recall for words. It confirms the picture superiority effect usually found and explained by the dual coding theory (e.g., [57]). Importantly, the interaction between posture and manipulability was confirmed (*F*(1,67) = 9.65, p = .003, η_p_² = .13). However, the format of presentation did not interact either with manipulability (*F*(1,67) = 1.91, p = .17) or with posture (*F* < 1), and the three-way interaction was not significant (*F* < 1).

To sum up, this second experiment replicates the main result of the first one, showing that the interfering posture decreased the recall of manipulable objects but not of nonmanipulable ones. The absence of an interaction effect with the format of presentation suggests that, in both experiments, the hands behind the back posture interferes with long-term memory of manipulable objects at a semantic level rather than at a perceptual level. This is consistent with the Glenberg’s claim that the world is conceptualized in terms of possible actions [4], which implies that interfering with these possible actions interferes with memory.

## General discussion

In two experiments, we tested the idea that long-term memory and motor systems share processing resources. For this purpose, participants had to learn lists of manipulable and nonmanipulable objects while adopting different postures, their hands at rest on a desk as a control posture, or hands crossed behind their back as an interfering posture. In both studies, results consistently showed that recall for manipulable objects decreased with the interfering posture, while no effect of the interfering posture was found on nonmanipulable objects. These results are in line with previous findings showing that motor interference has a selective and negative effect on the processing of manipulable objects [38,41], and, more generally, on the processing of motor related concepts [58]. The interfering effect was similar regardless of the presentation format, pictures (Exp 1) or words (Exp 2), which is consistent with previous research [23,27]. This suggests that the effect observed is not exclusively driven by the visual properties of the objects. Instead, the interference occurred more likely at a semantic level. Therefore, this can be taken as a strong evidence for the idea that motor simulation has a functional role in the memory of concepts of manipulable objects.

The mechanism behind the interference produced by the hands behind the back posture remains to be better understood. As stated earlier, the processing of manipulable object concepts involves a motor simulation related to prior interactions with the object, that is, related essentially to its function [56]. Consequently, the episodic memory of the object should include this particular motor component. One possible way to explain our results is to assume that the interfering posture inhibits the motor area responsible for the motor simulation in order to inform the organism that a manual action is not possible in such a body state. This inhibition may then reduce the memory trace of the object. There are actually some evidences that posture can modulate the use of motor information. In particular, it seems that posture affect motor imagery performances [51,59–61]. For instance, Sirigu and Duhamel [51] have shown that motor imagery response times are slower with the hands behind the back than with the hands on the desk. Consistently, as shown in a TMS experiment, a posture incompatible with the action to be imagined decreases the excitability of the motor cortex [62]. As suggested by this literature, the use of other techniques may be fruitful to investigate the nature of the present postural effect. For instance, the effect of posture on cortical motor activity when a manipulable object is presented could be studied using TMS or neuroimaging.

Then, the interference observed on memory with the hands behind the back may be caused by several features of this posture. The interference may arise from the fact that the hands are constrained behind the back, and not in front. It assumes that any interfering posture or task with the hands in front of the body would not create the same interference. More generally, it may also arise from the physical constraint induced by the posture which makes the body unavailable for action. If so, any constraining posture should produce the same effect, even if the hands are in front of the body. Instead of being postural, the effect could also come from the knowledge about the constraint, which implies that tying the hands in front of the participant should be sufficient to replicate the present results.

Finally, one can wonder whether our experiments succeeded in showing that interfering with the motor system with a constraining posture implied a deficit in memory, since there are reasons for interpreting the observed effect as a facilitation due to the hands on the desk posture, rather than an interference caused by the hands behind the back posture. Indeed, the present findings might be related to a retrieval mechanism, and more precisely to the encoding specificity principle, rather than to an encoding mechanism as hypothesized. The encoding specificity principle states that memory is better when the contexts during encoding and retrieval are similar [63]. It posits that memory performances should be higher when the posture during encoding is the same as during retrieval, as in the control condition. Thus, instead of resulting from an interference of the hands behind the back posture during encoding, the present findings could result from a facilitation of the congruent posture during recall. However, the fact that this encoding specificity effect with posture works only for manipulable objects still needs to be explained. A possible interpretation is that the motor cue is relevant for manipulable objects but not for nonmanipulable ones. A different posture at retrieval could make it more difficult for the system to use the motor cue and reinstate the encoded motor simulation. Yet, this interpretation is still in line with the idea that memory of action related concepts, i.e. manipulable objects, depends on the actual motor state of the organism. Therefore, it still advocates for an interpretation in terms of shared motor properties between the posture and the objects, which influence the memory process. Therefore, this work can be taken as strong evidence for an embodied account of language and semantic memory (e.g., [1,3]).

To conclude, this research newly shows that motor interference, i.e. the hands behind the back posture, decreases the memory of manipulable objects, suggesting that the motor simulation has a role in the long-term semantic representation of these objects. Thus, this work shows quite clearly that long-term memory and motor systems share resources, and supports Glenberg’s view that memory is for guiding action.

## Supporting Information

S1 File List of the French words and their English translation for the manipulable (Appendix A in S1 File) and nonmanipulable (Appendix B in S1 File) objects. Words are presented together with their length, frequencies and imageability.
(DOCX)
