# Supplementary material for: Learning Is Better with the Hands Free: The Role of Posture in the Memory of Manipulable Objects
Source: PLoS One. 2016 Jul 14;11(7):e0159108. doi: 10.1371/journal.pone.0159108 (PMC4944978; doi:10.1371/journal.pone.0159108)
Supplement: S1 File — Words are presented together with their length, frequencies and imageability. (DOCX) [file pone.0159108.s001.docx]

**Appendix A.** List of the French words and their English translation for the manipulable objects used in Experiment 1 and 2 together with their length, frequencies and imageability. Objective frequencies are from New et al. (2001), and all subjective frequencies and imageability ratings are from Desrochers & Thompson (2009), except ^a^ from Bonin et al. (2011) and ^b^ from Desrochers & Bergerons (2000).

| Objects | Translation | Length | Objective frequency | Subjective frequency | Imageability |
| --- | --- | --- | --- | --- | --- |
| agrafeuse | stapler | 9 | 0.07 | 3.26 | 6.52 |
| allumette | matchstick | 9 | 9.73 | 5.29 ^b^ | 6.78 ^b^ |
| Arrosoir | watering can | 8 | 3.04 | 3.27 | 6.39 |
| Balai | broom | 5 | 11.96 | 4.40 | 6.67 |
| bouilloire | kettle | 10 | 3.45 | 4.25 | 6.40 |
| Bouteille | bottle | 9 | 70.41 | 5.69 | 6.64 |
| Briquet | lighter | 7 | 12.30 | 3.59 ^b^ | 6.67 ^b^ |
| Brosse | brush | 6 | 16.01 | 5.19 | 6.25 |
| Canne | cane | 5 | 26.62 | 3.46 ^a^ | 6.20 ^a^ |
| Carafe | jug | 6 | 4.46 | 1.98 | 3.78 |
| casserole | saucepan | 9 | 16.22 | 4.29 ^b^ | 6.33 ^b^ |
| Ciseaux | scissors | 7 | 11.01 | 4.98 | 6.87 |
| Clé | key | 3 | 35.00 | 5.62 | 6.63 |
| Couteau | knife | 7 | 44.26 | 5.42 | 6.58 |
| Crayon | pencil | 6 | 25.47 | 5.45 | 6.67 |
| Cuillère | spoon | 8 | 9.80 | 5.73 | 6.82 |
| Feutre | felt pen | 6 | 13.18 | 4.11 ^a^ | 6.53 ^a^ |
| fourchette | fork | 10 | 10.95 | 5.26 | 6.87 |
| Gomme | eraser | 5 | 9.26 | 4.61 ^a^ | 6.73 ^a^ |
| Marteau | hammer | 7 | 13.31 | 4.12 | 6.85 |
| parapluie | umbrella | 9 | 12.50 | 4.54 | 6.77 |
| Peigne | comb | 6 | 8.85 | 4.85 | 6.75 |
| Pelle | shovel | 5 | 11.35 | 4.14 | 6.54 |
| Pince | clamp | 5 | 7.64 | 3.75 ^a^ | 6.07 ^a^ |
| Pinceau | paintbrush | 7 | 10.27 | 3.84 | 6.60 |
| Pistolet | gun | 8 | 14.80 | 2.73 | 6.55 |
| Poêle | frying pan | 5 | 17.84 | 5.24 ^a^ | 6.73 ^a^ |
| Raquette | racket | 8 | 1.69 | 4.46 ^b^ | 6.56 ^b^ |
| Rasoir | shaver | 6 | 15.61 | 4.75 | 6.43 |
| Râteau | rake | 6 | 1.62 | 3.09 | 6.47 |
| Scie | saw | 4 | 8.11 | 3.38 | 6.53 |
| Seau | bucket | 4 | 14.73 | 3.32 ^a^ | 5.83 ^a^ |
| Stylo | pen | 5 | 10.61 | 4.80 | 6.70 |
| Tasse | cup | 5 | 25.07 | 5.28 | 6.85 |
| téléphone | phone | 9 | 93.99 | 6.36 | 6.80 |
| tournevis | screwdriver | 9 | 3.24 | 3.90 | 6.78 |
| Mean |  | 6.75 | 16.79 | 4.40 | 6.50 |
| Standard-Error |  | 0.31 | 3.12 | 0.16 | 0.09 |

**Appendix B.** List of the French words (and their English translation) for the nonmanipulable objects used in Experiment 1 and 2 together with their length, frequencies and imageability. Objective frequencies are from New et al. (2001), and all subjective frequencies and imageability ratings are from Desrochers & Thompson (2009), except ^a^ from Bonin et al. (2011) and ^b^ from Desrochers & Bergerons (2000).

| Objects | Translation | Length | Objective frequency | Subjective frequency | Imageability |
| --- | --- | --- | --- | --- | --- |
| Antenne | antenna | 7 | 3.65 | 3.42 ^b^ | 6.35 ^b^ |
| Avion | plane | 5 | 46.82 | 4.83 | 6.76 |
| baignoire | bathtub | 9 | 14.12 | 3.34 ^b^ | 6.76 ^b^ |
| balançoire | swing | 10 | 1.89 | 3.68 | 6.59 |
| Banc | bank | 4 | 48.31 | 4.57 ^b^ | 6.44 ^b^ |
| Barrière | fence | 8 | 17.36 | 4.08 | 5.70 |
| Bateau | boat | 6 | 61.22 | 4.63 | 6.84 |
| Bus | bus | 3 | 10.54 | 5.64 ^a^ | 6.90 ^a^ |
| Cabane | hut | 6 | 25.68 | 5.11 ^b^ | 6.43 ^b^ |
| Camion | truck | 6 | 30.27 | 5.33 | 6.73 |
| Canapé | couch | 6 | 17.97 | 2.78 | 5.63 |
| Château | castle | 7 | 63.38 | 3.72 | 6.84 |
| cheminée | chimney | 8 | 36.28 | 4.12 | 6.70 |
| Cloche | bell | 6 | 18.24 | 4.16 | 6.52 |
| Eglise | church | 6 | 123.58 | 4.55 | 6.55 |
| Fauteuil | armchair | 8 | 76.69 | 5.13 ^b^ | 6.81 ^b^ |
| Fontaine | fountain | 8 | 17.36 | 3.85 ^b^ | 6.60 ^b^ |
| Frigo | fridge | 5 | 7.85 | 4.33 | 6.74 |
| Grue | crane | 4 | 2.84 | 2.16 | 5.11 |
| guirlande | garland | 9 | 2.30 | 3.76 ^b^ | 6.27 ^b^ |
| Horloge | clock | 7 | 13.99 | 5.01 | 6.84 |
| immeuble | building | 8 | 50.88 | 4.29 | 5.38 |
| lampadaire | street light | 10 | 2.84 | 3.01 | 6.24 |
| Miroir | mirror | 6 | 48.58 | 5.43 | 6.78 |
| Moulin | windmill | 6 | 15.61 | 3.02 | 6.23 |
| Panneau | panel | 7 | 16.55 | 3.76 | 5.72 |
| Phare | lighthouse | 5 | 10.68 | 3.43 ^a^ | 6.37 ^a^ |
| Pneu | tire | 4 | 4.93 | 3.93 ^a^ | 6.57 ^a^ |
| Puits | well | 5 | 21.69 | 2.95 | 5.56 |
| Radiateur | radiator | 9 | 6.35 | 2.96 ^b^ | 6.16 ^b^ |
| Tabouret | stool | 8 | 15.47 | 3.16 ^b^ | 5.99 ^b^ |
| Tapis | carpet | 5 | 60.88 | 5.62 ^b^ | 6.76 ^b^ |
| télévision | television | 10 | 23.51 | 6.26 ^b^ | 6.83 ^b^ |
| Tonneau | barrel | 7 | 6.89 | 2.69 | 5.13 |
| Tracteur | tractor | 8 | 5.27 | 3.77 ^b^ | 6.61 ^b^ |
| ventilateur | fan | 11 | 2.09 | 2.97 ^b^ | 6.02 ^b^ |
| Mean |  | 6.86 | 25.90 | 4.04 | 6.35 |
| Standard Error |  | 0.32 | 4.43 | 0.16 | 0.09 |
